# Development and efficacy of a psychoeducational family intervention for perinatal depression

**DOI:** 10.1192/j.eurpsy.2021.76

**Published:** 2021-08-13

**Authors:** A. Fiorillo

**Affiliations:** Department Of Psychiatry, University of Campania “L. Vanvitelli”, Naples, Italy

## Abstract

**Abstract Body:**

The perinatal period represents an at-risk period for mental health consequences, which has been overlooked for long time. Perinatal mental health problems constitute a relevant threat for long-term mental health, not only for the direct impact on the affected women, but also for the considerable foetal/infant morbidity and mortality. Perinatal mental disorders are associated with negative outcomes in the newborn, including an increased risk of premature delivery and infant mortality, as well as a higher prevalence of mental disorders in the offspring (e.g., attention deficit or anxiety disorders). Depressive disorders represent the most common disorder during the perinatal period. For the adequate, appropriate and complete management of women with perinatal depression, there is the need for integrated interventions, following a comprehensive global assessment of women’s mental health. In particular, the management of depression during perinatal period requires special attention, even considering the problems and limitations in prescribing pharmacological drugs. In this context, psychoeducational interventions are effective in reducing affective symptoms and the levels of stress, with low costs for the mental health department.

**Disclosure:**

No significant relationships.

